# Discovery of a specific inhibitor of human GLUT5 by virtual screening and *in vitro* transport evaluation

**DOI:** 10.1038/srep24240

**Published:** 2016-04-14

**Authors:** Alayna M. George Thompson, Oleg Ursu, Petr Babkin, Cristina V. Iancu, Alex Whang, Tudor I. Oprea, Jun-yong Choe

**Affiliations:** 1 Department of Biochemistry and Molecular Biology, Rosalind Franklin University of Medicine and Science, The Chicago Medical School, 3333 Green Bay Road, North Chicago, IL, 60064, USA; 2 Department of Internal Medicine, Translational Informatics Division, MSC09 5025, University of New Mexico School of Medicine, Albuquerque, NM 87131, USA

## Abstract

GLUT5, a fructose-transporting member of the facilitative glucose transporter (GLUT, SLC2) family, is a therapeutic target for diabetes and cancer but has no potent inhibitors. We virtually screened a library of 6 million chemicals onto a GLUT5 model and identified N-[4-(methylsulfonyl)-2-nitrophenyl]-1,3-benzodioxol-5-amine (MSNBA) as an inhibitor of GLUT5 fructose transport in proteoliposomes. MSNBA inhibition was specific to GLUT5; this inhibitor did not affect the fructose transport of human GLUT2 or the glucose transport of human GLUT1-4 or bacterial GlcP_Se_. In MCF7 cells, a human breast cancer cell line, MSNBA competitively inhibited GLUT5 fructose uptake with a K_I_ of 3.2 ± 0.4 μM. Ligand docking, mutagenesis and functional studies indicate that MSNBA binds near the active site and inhibitor discrimination involves H387 of GLUT5. Thus, MSNBA is a selective and potent inhibitor of fructose transport via GLUT5, and the first chemical probe for this transporter. Our data indicate that active site differences in GLUT members could be exploited to further enhance ligand specificity.

Fructose is one of the most common dietary carbohydrates. During our evolution, humans have consumed relatively small amounts of fructose mostly from fruits. Recent studies indicate that fructose consumption increased by almost 50% among US adults in the last 40 years, with fructose accounting for at least 10% of daily calories on average^1^. Unlike glucose, fructose in serum is not regulated by insulin, and high levels of fructose consumption can cause dyslipidemia, impair glucose homeostasis and increase insulin resistance^2^. Some studies also link a fructose-rich diet with hypertension^3^,^4^.

Fructose transport across cell membranes is carried out by members of the facilitated glucose transporter (GLUT, SLC2) family. Among the 14 members of human GLUT protein family, only GLUT5 is fructose specific and lacks the ability to transport other carbohydrates such as glucose and galactose^5^,^6^,^7^. GLUT5 is expressed in intestinal epithelia, sperm, brain, fat, skeletal muscle and kidney cells^8^.

Cancer cells require more energy for their uncontrolled growth and usually exhibit increased rates of carbohydrates transport, compared to normal cells. GLUT5 is frequently overexpressed in cancer cells (~27% of analyzed tumors)^9^. For example, GLUT5 is not normally present in mammalian breast cells, but the breast carcinoma cell lines MCF7 and MDA-MB-231 exhibit elevated GLUT5 mRNA level and show high rates of fructose transport^10^. Additionally, *in vitro* studies linked fructose exposure to modification of the glycan structures on the cell surface that enhance cancer cell invasiveness and proliferation^11^. Also, abolishing GLUT5 expression in breast cancer cells inhibited tumor proliferation^12^. Pancreatic cancer cells prefer fructose in their nucleic acid synthesis, thus fructose promotes pancreatic cancer proliferation^13^. Consequently, GLUT5 activity is being explored as a marker for cancer, and development of fluorinated fructose analogs for use in positron emission tomography (PET) cancer diagnosis in GLUT5-overexpressing tumors is underway^14^.

In normal tissue, the expression of GLUT5 is upregulated by fructose^15^ and may be the underlying mechanism linking GLUT5 with metabolic disorders^8^. GLUT5 is upregulated in some diabetic patients and this expression is reversible with diabetes management treatment^16^.

Given the medical importance of GLUT5, its inhibitors could potentially serve as therapeutics for the treatment and management of cancer or diabetes. Nonetheless, selective and potent inhibitors for GLUT5 have not been described. Despite the high sequence similarity among GLUT members, known inhibitors of other GLUT proteins (for example cytochalasin B, phloretin, or forskolin) do not affect GLUT5, suggesting that subtle differences may be responsible for ligand specificity among GLUT family members (this work,^17^,^18^). Indeed, a recent study found two natural products that inhibit GLUT5; one of which, rubusoside, a natural sweetener from the Chinese sweet tea plant (*Rubus suavissimus*), inhibited both GLUT1 and GLUT5, the first example of a common inhibitor for these two GLUTs. The other molecule, astragalin-6-glucoside, a flavonoid compound from the American pokeweed (*Phytolacca americana*) did not affect GLUT1. These inhibitors, though not potent (IC_50_ values ~ 5 mM) allowed for the identification of active site differences between GLUT1 and GLUT5 responsible for ligand specificity^17^.

Viable drugs targeting the fructose transport of GLUT5 should not interfere with transport mediated by other GLUT proteins. For instance, disrupting glucose transport by the insulin-dependent GLUT4 can lead to diabetes^19^. GLUT1, another transporter of glucose, is expressed in most tissues, so its inhibition can cause serious side effects at the organism level. Ideally, GLUT5 specific inhibitors should only affect GLUT5 mediated fructose transport, without altering aspects of metabolism unrelated to fructose consumption.

In this work we describe a combination of *in silico* and *in vitro* experiments that determined a specific inhibitor of human GLUT5. We screened *in silico* a library of six million compounds for binding to a GLUT5 model. The top ranked 175 *in silico* hits were tested for inhibition of fructose transport by human GLUT5 in proteoliposomes, and we found that N-[4-(methylsulfonyl)-2-nitrophenyl]-1,3-benzodioxol-5-amine (MSNBA, SMILES: [S](=O)(=O)(C)c1cc(c(cc1)Nc2cc3c(cc2)OCO3)[N+](=O)[O−]) inhibited GLUT5. Additionally, MSNBA potently inhibited fructose uptake by GLUT5 in the human breast adenocarcinoma cell line MCF7.

MSNBA did not affect the transport activity of human GLUT1-4 or GlcP_Se_, appearing to be specific for GLUT5. Docking of MSNBA to the GLUT5 model, along with mutagenesis and functional studies on GLUT5, GLUT1 and GlcP_Se_, suggested that the inhibitor bound close to the active site and pinpointed a GLUT5-specific His residue as a key determinant of MSNBA recognition. MSNBA together with the active site differences between GLUT5 and GLUT1 highlighted by MSNBA inhibition can be exploited for the rational design of potent, specific inhibitors for GLUT5 that will aid in cancer or diabetes treatment. Additionally, MSNBA provides the first GLUT5-specific chemical probe that can be used to investigate the role of fructose uptake in mechanisms of these diseases.

## Results

### *In silico* screening for potential GLUT5 inhibitors

The model of GLUT5 was initially generated on the basis of the inward-facing GlcP_Se_ crystal structure (PDB ID 4LDS) with Coot^20^. GlcP_Se_, a glucose/H^+^ symporter from *Staphyloccocus epidermidis*, is a bacterial GLUT homologue that shares 30% amino acid sequence identity with human GLUT5^21^. After the virtual screening studies were completed, the crystal structures of human GLUT1^22^ and bovine GLUT5^23^ were published and we generated new GLUT5 models on the basis of these new structures [PDB IDs: 4PYP (hGLUT1) and 4YB9 (bGLUT5)]. There is significant variation in the soluble loops depending upon the starting model, but the transmembrane substrate cavity in all GLUT5 homology models was invariable, consistent with the similarity of the crystal structures (Supplementary Fig. S1); the root-mean-square deviations for the superposition in the transmembrane helices was less than 1.5 Å for hGLUT5 models constructed from GlcP_Se_, hGLUT1 and bGLUT5 structures, as calculated with Superpose^24^. Our modeled GLUT5 was screened against Chemnavigator’s (http://www.chemnavigator.com) library of 6,273,384 small molecular compounds, and a list of 374 possible GLUT5 ligands was generated. Among these, considering commercial availability and cost, 175 were purchased and tested for inhibition of GLUT5 transport activity (Supplementary Table S1).

### *In vitro* screening for specific inhibitors of GLUT5

**Figure 1:**
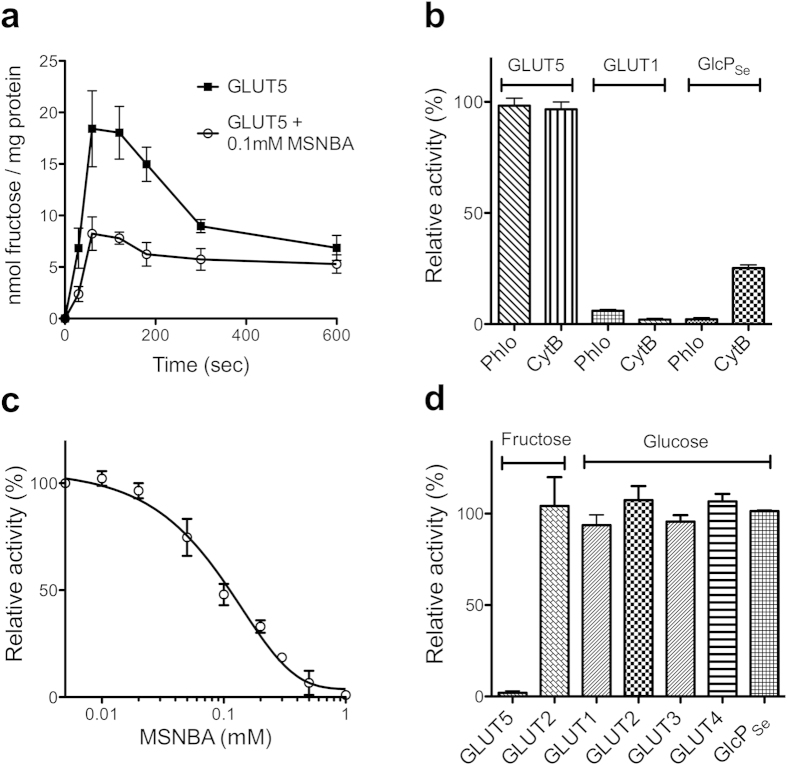
Effect of MSNBA on human GLUT1-5 and GlcP_Se_ transport activities. (**a**) Fructose entrance counter-flow transport in GLUT5 proteoliposomes, in the absence (filled squares) and presence (empty circles) of 0.1 mM MSNBA. Transport was initiated by the addition of GLUT5 proteoliposomes to the reaction solution containing 10 μM C^14^-fructose and stopped at the indicated time points. (**b**) Effect of common GLUT1 inhibitors on the relative transport activity of GLUT5, GLUT1 and GlcP_Se_. Glucose (for GLUT1) or fructose (for GLUT5) entrance counter-flow transport was measured one minute after initiation of transport by adding proteoliposomes to assay solution containing 10 μM C^14^-hexose and 2 mM phloretin (Phlo) or cytochalasin B (CytB). Glucose uptake for GlcP_Se_ was measured one minute after initiation of transport with 30 μM C^14^-glucose in the presence of 2 mM phloretin or cytochalasin B in right-side-out vesicles. (**c**) Dose-dependent MSNBA inhibition of GLUT5 fructose transport in proteoliposomes. Each point was measured one minute after transport initiation, using the entrance counter-flow transport assay. IC_50_ of MSNBA inhibition was 0.10 ± 0.03 mM. Curve was calculated with Prism (GraphPad Software). (**d**) Effect of 2 mM MSNBA on the fructose transport by GLUT5 or GLUT2 or glucose transport by GLUT1-4 or GlcP_Se_. GLUT1-5 transport activity was measured in proteoliposomes, using the entrance counter-flow assay, as in (**b**). GlcP_Se_ glucose transport was measured in right-side-out vesicles as in (**b**). Error bars represent standard deviations from 3 different experiments (**a–d**).

Human GLUT5 was recombinantly expressed in insect cells, purified, and reconstituted in artificial lipids as previously reported^17^. The fructose entrance counter-flow transport with GLUT5 proteoliposomes was constant between 1 and 2 minutes, so the inhibition of transport by compounds was measured one minute after assay initiation (Fig. 1a). Consistent with previous reports, GLUT5 transport was not affected by classical inhibitors of GLUT1 such as phloretin^25^ or cytochalasin B^10^ (Fig. 1b). The 175 potential GLUT5 ligands (Supplementary Table S1) were grouped in batches of 5 chemicals (each at 1 mM concentration) and tested for inhibition of fructose entrance counter-flow transport by GLUT5 in proteoliposomes. For batches that caused decreased fructose transport, the individual compounds from the group were tested for inhibition.

We found one chemical with inhibitory activity against GLUT5: N-[4-(methylsulfonyl)-2-nitrophenyl]-1,3-benzodioxol-5-amine (MSNBA) (Fig. 1a,c,d; the chemical structure of MSNBA is shown in Fig. 3b). MSNBA inhibited GLUT5 mediated fructose transport with an IC_50_ of 0.10 ± 0.03 mM (Fig. 1c); this is 100-fold lower than the reported fructose K_M_ for GLUT5 (~10 mM)^10^.

Given the desirability of a selective inhibitor for GLUT5, we investigated whether MSNBA inhibits GLUT1-4 or GlcP_Se_. Among these GLUT1, GLUT3, GLUT4 and GlcP_Se_ transport glucose, while GLUT2 transports both glucose and fructose^6^. Glucose transport of GLUT1, GLUT2, GLUT3 and GLUT4 or fructose transport of GLUT2 were assayed by the entrance counter-flow method in proteoliposomes loaded with the respective recombinant human GLUT. Glucose transport of GlcP_Se_ was measured in right-side-out (RSO) vesicles from JM1100 *E. coli* cells (that lack endogenous glucose transport) expressing GlcP_Se_. High concentrations of MSNBA (2 mM) did not influence the glucose transport of GLUT1, GLUT2, GLUT3, GLUT4 or GlcP_Se_ or the fructose transport of GLUT2 but completely abolished GLUT5 fructose transport (Fig. 1d).

The bioactivity profile of MSNBA was evaluated using three different online resources: i) PubChem (https://pubchem.ncbi.nlm.nih.gov), which has an entry for MSNBA (CID 4783927), does not show recorded bioactivities for this compound; ii) ChEMBL 21 (https://www.ebi.ac.uk/chembl/), and SureChEMBL (https://www.surechembl.org), have no entries for MSNBA. The on-line chemical portal, ChemSpider (https://www.chemspider.com), does not have additional information compared to PubChem. These searches indicate that there is no patented or published bioactivity profile for MSNBA, which further confirms our observations that this compound is highly specific for GLUT5.

### MSNBA inhibition of fructose uptake in MCF7 cells

**Figure 2:**
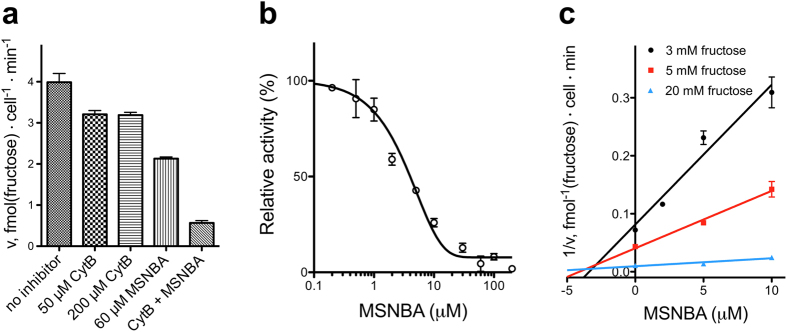
Inhibition of fructose uptake in MCF7 cells by MSNBA. (**a**) Inhibitor effect on fructose uptake into MCF7 cells. Cytochalasin B (50 or 200 μM) and MSNBA (60 μM) individually or in combination (CytB + MSNBA is 50 μM cytochalasin B and 60 μM MSNBA), were pre-incubated with MCF7 cells for 5 minutes. The uptake assay was initiated by the addition of 10 mM fructose and the transport was stopped after 30 minutes. (**b**) Dose-dependent MSNBA inhibition of fructose uptake after 30 minutes in MCF7 cells in the presence of 10 mM fructose and 50 μM cytochalasin B. IC_50_ of MSNBA inhibition was 5.8 ± 0.5 μM. (**c**) Dixon plot of fructose uptake after 2 minutes into MCF7 cells with varying fructose and MSNBA concentrations, in the presence of 50 μM cytochalasin B. MSNBA displays competitive inhibition of fructose uptake with K_I_ = 3.2 ± 0.4 μM. Kinetic parameters were determined with Prism (GraphPad Software).

To evaluate MSNBA inhibition of native human GLUT5, we measured the effect of MSNBA on the fructose uptake in cultured breast cancer cells MCF7, which express GLUT5^10^. Besides GLUT5, MCF7 cells also express GLUT1 and GLUT2^9^. Among these, only GLUT2 and GLUT5 are capable of fructose uptake. Cytochalasin B inhibits GLUT2 potently (K_I_ of ~6 μM) but does not affect or bind GLUT5^18^,^26^,^27^. Cytochalasin B at 50 or 200 μM had a moderate effect on fructose transport in MCF7 cells, while MSNBA by itself or in combination with cytochalasin B exhibited additional inhibition of fructose uptake (Fig. 2a). Therefore, to isolate the fructose uptake due to GLUT5, 50 μM of cytochalasin B was included in all uptake assays. We also measured glucose uptake into MCF7 cells (Supplementary Fig. S2) and found that MSNBA had no effect on glucose uptake while cytochalasin B reduced it by 20%.

In the presence of 10 mM fructose (reported K_M_ for human GLUT5^10^), MSNBA inhibited fructose uptake in MCF7 cells with an IC_50_ of 5.8 ± 0.5 μM (Fig. 2b). This is ~20 fold lower than the IC_50_ of MSNBA inhibition of the entrance counter-flow transport of fructose in GLUT5 proteoliposomes (Fig. 1c). The entrance counter-flow assay requires a high concentration of substrate (200 mM) and involves substrate exchange, not uptake. So it is likely that the counter-flow assay overestimates the inhibitor IC_50_.

To determine the mode of competition by MSNBA, initial velocity of uptake was measured by stopping the assay after two minutes (Supplementary Fig. S3). Analysis of inhibition of GLUT5 fructose uptake in MCF7 cells at different fructose and MSNBA concentrations shows that MSNBA is a competitive inhibitor of fructose transport with a K_I_ of 3.2 ± 0.4 μM (Fig. 2c).

### Determination of MSNBA binding site in GLUT5 model

GLUT proteins have 12 transmembrane (TM) helices organized into two domains (each of 6 helices, called the N- and C-domains), related by a two-fold pseudosymmetry axis. The substrate binding site is housed in the central cavity formed by the N- and C-domains^21^,^22^,^28^. The transport mechanism of GLUT proteins, similarly to other major facilitator superfamily (MFS) carriers, involves the alternating opening and closing of the substrate binding site to each side of the membrane. Consistent with this mechanism, available crystal structures of sugar porters capture the inward-facing (GlcP_Se_^21^, human GLUT1^22^ and bovine GLUT5^23^; PDB IDs 4LDS, 4PYP and 4BY9, respectively), outward-occluded (XylE^28^ and human GLUT3^29^; PDB IDs 4GBY and 4ZWB, respectively) and outward-open (human GLUT3^29^ and rat GLUT5^23^; PDB IDs 4ZWC and 4YBQ, respectively) conformations. The GLUT5 model used for virtual screening was based on the GlcP_Se_ crystal structure, which is in the inward-facing conformation.

**Figure 3:**
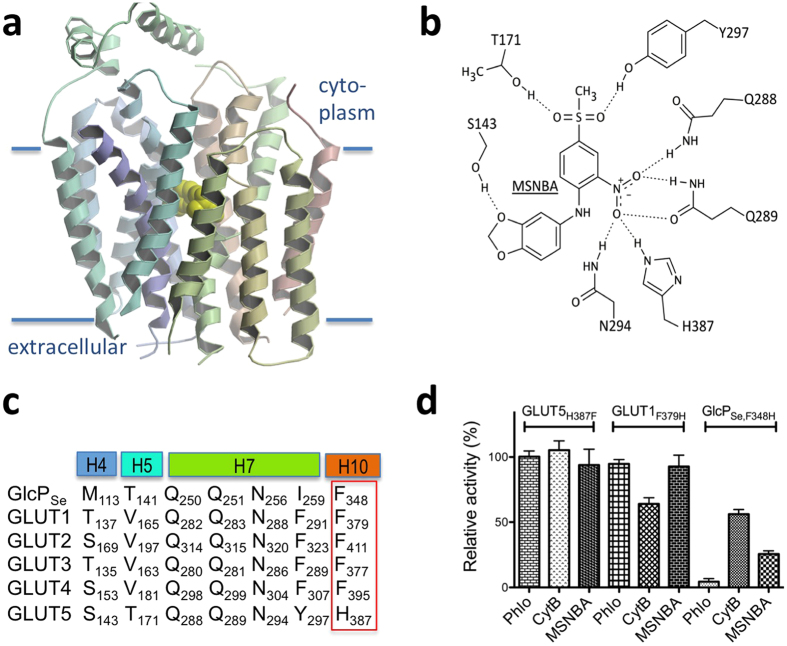
Modeled interaction of MSNBA with GLUT5. (**a**) Binding of MSNBA (yellow CPK model) to the transmembrane site in GLUT5 as predicted by docking of the inhibitor to GLUT5 model with MOE. The figure was drawn with Molscript^46^ and raster3D^47^. (**b**) Interactions between MSNBA and side chains of GLUT5. Interacting residues come from helices 4 (S143), 5 (T171), 7 (Q288, Q289, N294 and Y297), and 10 (H387); see also Supplementary Fig. S4. (**c**) Multiple sequence alignment among GLUT homologues for the GLUT5 residues predicted to interact with MSNBA. H4, H5, H7 and H10 represent transmembrane helices 4, 5, 7 and 10, respectively, and are color-coded as in Fig. 3a. The red box highlights the residue explored by site-directed mutagenesis in GLUT1, GlcP_Se_ and GLUT5. Alignment was generated with ClustalW^48^. (**d**) Effect of MSNBA and common GLUT1 inhibitors cytochalasin B (CytB) and phloretin (Phlo) on the relative transport activity of GLUT5_H387F_, GLUT1_F379H_ or GlcP_Se,F348H_ measured as in Fig. 1b except that for GlcP_Se,F348H_ glucose concentration was 90 μM (corresponding to glucose K_M_ for this mutant, Supplementary Fig. S5). All inhibitors were at 2 mM concentration.

The docked MSNBA binding site in the GLUT5 model is near the transmembrane active site (Fig. 3a). MSNBA putatively interacts with residues from the N-domain including S143 (helix 4) and T171 (helix 5), as well as the C-domain residues (Q288, Q289, N294, Y297) from helix 7, and H387 from helix 10 (Fig. 3b and Supplementary Fig. S4). These residues vary in their degree of conservation among human GLUT proteins or GlcP_Se_ (Fig. 3c). Q288, Q289 and N294 are strictly conserved and essential for glucose transport as their mutations abolish transport activity in GLUT1^30^ or GlcP_Se_^21^. S143 and T171 are not systematically conserved. There are two residues that are different in GLUT5 compared to glucose transporters: Y297 is a Phe in GLUT1-4 or Ile in GlcP_Se_, while H387 is Phe in GLUT1-4 and GlcP_Se_ (Fig. 3c). GLUT1, GLUT3, GLUT4 and GlcP_Se_ transport glucose but not fructose, GLUT2 transports both fructose and glucose, while GLUT5 transports only fructose^7^.

As MSNBA seemed to be specific for GLUT5, we focused on the residues in the MSNBA predicted binding site that were different in GLUT5 compared to GLUT1-4 and GlcP_Se_. Among these, H387 of GLUT5 potentially interacts with the nitro group of MSNBA (Fig. 3b and Supplementary Fig. S4), but the Phe present in GLUT1-4 as well as GlcP_Se_ would have reduced interaction with MSNBA (Fig. 3c). To test if this residue is important for MSNBA inhibition, we constructed and expressed three mutant proteins: GlcP_Se,F348H_, GLUT1_F379H_ and GLUT5_H387F_. As with the wild-type proteins, GLUT1_F379H_ and GLUT5_H387F_ were recombinantly produced in insect cells, purified, reconstituted in proteoliposomes, and then activity was assayed by the entrance counter-flow method in proteoliposomes. Glucose uptake by GlcP_Se,F348H_ was measured in RSO vesicles of JM1100 *E. coli* cells. Unlike in wild-type transporters, 2 mM MSNBA inhibited 75% of the transport activity of GlcP_Se,F348H_ (Fig. 3d), and left GLUT5_H387F_ largely unaffected (Fig. 3d). Nevertheless, GLUT1_F379H_ remained insensitive to MSNBA, similarly to wild-type GLUT1 (Figs 1b and 3d).

Interestingly, the susceptibility of GLUT1_F379H_ to the well-known GLUT1 inhibitors cytochalasin B and phloretin (Fig. 3d) was different from that of the wild-type GLUT1 (Fig. 1b), with the mutant becoming completely resistant to phloretin and partially resistant to cytochalasin B. GlcP_Se,F348H_ was also partially resistant to cytochalasin B inhibition but retained sensitivity to phloretin (Fig. 3d).

## Discussion

The investigation of GLUT5 involvement in diseases and its use as a therapeutic target could be greatly advanced by discovery of potent and specific inhibitors of its activity. Previously reported GLUT5 inhibitors are substrate analogs of low potency^17^. To determine novel ligands for GLUT5 we used a convergent virtual and *in vitro* screening approach that has previously allowed us to identify several “first-in-class” bioactive molecules: small molecule antagonists for the formyl peptide receptors; the first agonist^31^ for the G-protein estrogen receptor (GPER, or GPR30), “G1”, and the first GPER antagonist^32^, “G15”; a selective ABCG2 transporter inhibitor^33^; and two HIV integrase inhibitors, Raltegravir and Elvitegravir, that block metnase, a DNA-repair enzyme^34^.

We virtually screened a model of GLUT5 in an inward-facing conformation. Because of the conformational cycling undergone by MFS proteins, it is possible that inhibitors could bind or stabilize one conformation and lock the protein. Thus, virtual screens using one conformation may not identify all possible ligands. There are now crystal structures of GLUT5 homologs in outward-facing conformations^23^, and efforts to identify new ligands binding to these models are on-going in our labs.

Our virtual screening procedure follows a previously described workflow^35^, which centers on chemical structure standardization and preparation, followed by careful evaluation of the binding site. In this work, we virtually screened a chemical library of over 6 million compounds against a putative binding site model of GLUT5, and reduced the number of possible ligands to 374. Among these, 175 were tested for inhibition of GLUT5 activity *in vitro*, in proteoliposomes, with the entrance counter-flow transport method.

With this combination of virtual and *in vitro* screening we identified MSNBA as a selective inhibitor of fructose transport by GLUT5. MSNBA competitively inhibited fructose uptake by GLUT5 into MCF7 cells with a Ki of 3.2 ± 0.4 μM. *In silico* docking predicted that the MSNBA binding site is near the transmembrane substrate transit site; this prediction is consistent with the observed competitive inhibition. His 387 of GLUT5 may interact with the nitro group of MSNBA; this position aligns with Phe in glucose transporters and could be the molecular determinant of specificity for MSNBA. Indeed, mutants in this position in human GLUT5 (GLUT5_H387F_) and the bacterial glucose transporter GlcP_Se_ (GlcP_Se_,_F348H_) showed changes in MSNBA inhibition consistent with a key role of His 387 in MSNBA recognition. GLUT1_F379H_ remained insensitive to MSNBA inhibition, suggesting that other residues in the active site, besides His 387, are involved in binding MSNBA. However, the desensitization of GLUT1_F379H_ to inhibition by cytochalasin B and phloretin strongly supports a significant role of this position in ligand recognition in different GLUT members. Among all human GLUTs only GLUT7 has a histidine residue aligned with GLUT5 His 387. We speculate that MSNBA may also inhibit GLUT7 and, thus it could be used to investigate the biological role of this less-studied GLUT member.

Taken together the data presented here and previous work on GLUT1 and GLUT5 inhibitors^17^ converge to suggest that, despite the overall similarity of GLUT members, small differences in their active sites can significantly impact ligand selectivity. This brings hope that while the molecular determinants of substrate specificity in GLUT1 and GLUT5 remain unclear, new small molecule chemical probes that selectively modulate the activity of each transporter can be discovered. Such probes could be important in the *in vivo* studies for various pathologies.

MSNBA is the first GLUT5-selective chemical probe, which completely blocks GLUT5-mediated fructose uptake. MSNBA may become an important starting point in the rational design of novel therapeutics against obesity, diabetes and cancer. Fructose is linked to obesity and diabetes, as fructose consumption is correlated with metabolic disorders, particularly impaired glucose homeostasis, insulin resistance and dyslipidemia^2^. Blocking fructose uptake from the gut into blood serum, could ameliorate the metabolic symptoms associated with our modern diets.

But the largest potential use of MSNBA is in understanding fundamental processes in cancer. Although we have shown that MSNBA can inhibit fructose uptake into an immortalized cancer cell line over a short time, the long-term effect on cells has yet to be evaluated. MSNBA could halt fructose-dependent proliferation or inhibit the metabolic changes seen in fructose-utilizing cancers.

Finally, MSNBA will allow experiments exploring fructose and its link to disease states. From epidemiological studies, the correlations between fructose and several diseases have been implied, but the molecular mechanisms are poorly understood. This specific GLUT5 inhibitor opens up new doors to understand the crucial relationship between our food and health.

## Methods

### Virtual screening

The 3D model for GLUT5 was built with the Coot software^20^, on the basis of GlcP_Se_ crystal structure (PDB ID 4LDS), using amino acid replacement guided by sequence alignments. Homology models of human GLUT5 based upon either human GLUT1 (4PYP) or bovine GLUT5 (4YB9) were constructed upon publication of the structures; these models are virtually identical to the model based on GlcP_Se_ (Supplementary Fig. S1). GLUT5 model was superimposed to the liganded XylE crystal structure (PDB ID 4GC0) to identify possible binding regions for GLUT5 ligands. All amino acid residues in GLUT5 model interacting with bromo-glucose were labeled and used to define the docking binding pocket through the following procedure: fructose molecule was placed in GLUT5 model using coordinates from bromo-glucose of XylE structure, and flexible docking was performed with Molecular Operating Environment (MOE, Chemical Computing Group Inc.), so that both ligand (fructose) and binding pocket residues were allowed to be flexible. Only the top 5 ligand-transporter scoring poses were saved and used with FRED docking^36^, given the time constraints of flexible docking on the ChemNavigator library (http://www.chemnavigator.com). The library was prepared for virtual screening as follows. Standard clean up procedure was employed where chemical structures were checked for valence errors, salt and solvent removal. A total of 6,273,384 chemicals were selected for further processing. Standardized chemical structures were then used to calculate 2D extended connectivity fingerprints (ECFP) using an in-house implementation of algorithm described in literature^37^. Conformer generation with Omega^38^ prepared input for 3D shape and docking applications. 3D conformations were generated using Merck Molecular Force Field 94^39^ with 400 maximum number of conformers for each chemical structure. Shape and pharmacophore screening were performed with ROCS^40^ based on lead molecules described in literature^41^. FRED program^36^ was used to dock conformers generated with Omega to the GLUT5 model. Ranking of hits was done using a combo score with 20%, 60% and 20% weights given to ECFP, ROCS and FRED scores, respectively. A total of 347 top scoring hits were selected based on the combo score described above, out of which 175 chemicals (Supplementary Table S1) were purchased and tested in GLUT5 proteoliposomes for inhibition of the fructose entrance counter-flow transport.

### Protein expression and purification of wild-type and mutant GLUTs

cDNAs of GLUT1, 3, 4 and 5 were purchased from Open Biosystems (GE Healthcare). cDNA of GLUT2 was a gift from Prof. Graeme Bell at University of Chicago. Full length DNA was subcloned into pFastBac1 vector (Life Technologies) with a N-terminal hexahistidine tag. Bacmids were generated in DH10Bac *E. coli* cells (Life Technologies). Baculoviruses were produced using Cellfectin II Reagent and amplified in Sf21 insect cells (Life Technologies). Cells were maintained at 26 °C, and P1 (10^6^ pfu/mL) was collected from infected cells after 72 hours. Sf21 cells were propagated in HyClone SFX-Insect media (GE Healthcare), supplemented with 5% (v/v) fetal bovine serum (Biowest), 4.8 mM glutamine, antibiotics (100 units/mL Penicillin G and 100 μg/mL streptomycin sulfate) and amphotericin B (2.5 μg/mL). For recombinant protein expression, Sf21 cells in suspension culture at 2 × 10^6^ cells/ml were infected with P3 viral stock (10^8^ pfu/mL), at an MOI (multiplicity of infection) of 1.0 pfu/cell. Four days after viral infection, cells from 1 L culture were collected by centrifugation at 2,000 g and 25 °C. The cell pellet was resuspended in 120 mL of buffer A [50 mM sodium phosphate (NaPi) (pH 7.5), 5% (v/v) glycerol, 200 mM sodium chloride], with protease inhibitors (1 mM AEBSF, 10 μM E-64, 10 μM pepstatin A, 1 μM Aprotinin, 20 μM Bestatin, 20 μM Leupeptin), at 4 °C, and disrupted by sonication (Branson Ultrasonic). n-Dodecyl-β-D-maltopyranoside (DDM, EMD chemicals) was added to a final concentration of 1% (w/v) in the broken cell solution, and the mixture was incubated at 4 °C with stirring for 4 hours. The solubilized protein solution was clarified by ultracentrifugation at 200,000 g and 4 °C for 1 hour, and the supernatant was loaded onto the Ni-NTA resin (EMD Millipore). The column was washed with buffer containing 50 mM NaPi (pH 7.5), 500 mM NaCl, 5–20 mM imidazole, 5% (v/v) glycerol and 0.05% (w/v) DDM. GLUT1-5 were eluted with buffer A, containing 300 mM imidazole and 0.05% DDM (w/v). To generate DNA for mutant proteins, site-directed mutagenesis was performed on the pFastBac1 plasmid constructs of wild-type proteins and verified by DNA sequencing^42^. Mutant proteins were expressed and purified in the same manner as wild-type proteins with no modifications.

### Proteoliposome preparation

Proteoliposomes were generated according to published protocols^21^,^43^ with minor modifications. Liposomes were made from a 95%/5% mix of soy phosphatidylcholine and cholesterol (Avanti Polar Lipids). Prepared liposomes were destabilized with 4 mM Triton X-100 and mixed with purified protein in a 100:1 (w/w) ratio in 100 mM KPi (pH 7.5), 20% (v/v) glycerol, 200 mM glucose for GLUT1-4 and GLUT1_F379H_ or fructose for GLUT5 (wild-type and H387F mutant) and GLUT2. Detergent was removed by several additions of SM2 BioBeads (BioRad), followed by incubation overnight at 4 °C. After filtering out BioBeads, proteoliposomes were diluted with 100 mM KPi (pH 7.5), 200 mM glucose for GLUT1 (wild-type and F379H mutant) and GLUT2-4 or fructose for GLUT5 (wild-type and H387F mutant) and GLUT2, and then collected by ultracentrifugation at 200,000 g, for 1 h, at 4 °C. Proteoliposomes were resuspended in 100 mM KPi (pH 7.5), to an OD_600nm_ ~30.

### Entrance counter-flow transport assay for human GLUTs

For the entrance counter-flow transport in proteoliposomes, the assay was started by the addition of 5 μL proteoliposomes solution (OD_600nm_ ~30) to 200 μL assay solution (100 mM KPi buffer at pH 7.5), containing 10 μM ^14^C-radiolabeled glucose (for GLUT1 wild-type or F379H mutant and GLUT2-4) or fructose (for GLUT5 wild-type or H387F mutant and GLUT2) (Moravek Biochemicals). After one minute (or different time points as specified), the transport was stopped with ice-chilled quench buffer [0.1 M KPi (pH 5.5) and 0.1 M lithium chloride]. The solution was filtered onto a cellulose nitrate membrane filter (Whatman; 0.4 μm pore size), and the filter was washed three times with quench buffer. The membrane filter was placed into a vial filled with BioSafe II scintillation liquid (Research Products International Corp.), and radioactivity was quantified with LS 6500 scintillation counter (Beckman). Compounds tested for the inhibition study were purchased from Sigma Chemicals (Supplementary Table S1). Stocks of 100 mg/ml for each compound were made in either water or dimethyl sulfoxide (DMSO). Chemicals at 1 mM final concentration in the assay volume were screened for inhibition of GLUT5 fructose transport in proteoliposomes. Tested inhibitors were added 1 min prior to addition of proteoliposomes solution. Cytochalasin B (Enzo Life Sciences) and phloretin (Alfa Aesar) were dissolved in DMSO at stock concentrations of 100 mM. Kinetic parameters were determined by nonlinear algorithm plots supplied by Prism (GraphPad Software). DMSO up to 5% (v/v) concentration in the transport assay did not affect activity. Data is presented as relative activity normalized to radioactivity of no inhibitor added as 100% and empty liposomes as 0%.

### Hexose uptake in MCF7 cells

MCF7 cells were acquired from the American Type Culture Collection (ATCC HTB-22) and cultured in DMEM/F12 media (HyClone) supplemented with 10% (v/v) fetal bovine serum (Biowest) and 1% Pen/Strep (Lonza), and grown in a 37 °C/CO_2_ incubator. Cells were seeded into 12-well culture plates with 1 ml media/well and assayed 4 days after seeding (~200,000 cells per well). Before assays, media was removed and replaced with 300 μL phosphate-buffered saline (PBS). MSNBA and/or cytochalasin B in DMSO or DMSO control were added to MCF7 in PBS and pre-incubated 5 minutes at 37 °C. Then ^14^C-fructose (total concentration of cold and radioactive fructose of 3, 5, 10 or 20 mM, as indicated) or ^14^C-glucose (total concentration of cold and radioactive glucose of 10 mM) was added to each well and incubated at 22 °C for times indicated. Reaction was terminated by addition of 1 ml of ice-cold PBS. Cells were washed again with PBS and then lysed with 1% (w/v) Triton X-100 and 10 mM NaOH. Lysates were transferred to scintillation vial, diluted with 10 ml scintillation cocktail and measured with Becker LS 6500 Multi-purpose Scintillation Counter. Each measurement was the result of averaging 3 wells.

### Transport assay for GlcP_Se_

Glucose transport assay for GlcP_Se,_ wild-type and F348H mutant, was performed as described previously^21^. GlcP_Se_ was cloned into the pBAD vector (Invitrogen) with C-terminal 6xHis tag. F348H mutation was done with the site-directed mutagenesis method^42^ and verified by DNA sequencing. GlcP_Se_ proteins were expressed in the glucose transport deficient *Escherichia coli* strain JM1100 (the Coli Genetic Stock Center). Cells were grown at 37 °C, in Luria Broth medium, with 100 μg/mL ampicillin. Protein expression was induced with 0.3 mM L-arabinose at O.D._600nm_ 0.6. After 3 hours, cells were harvested by centrifugation at 2,500 g for 5 min. The right-side-out (RSO) membrane vesicles of JM1100 *E. coli* cells were prepared as described previously^21^,^44^,^45^. Transport assay was initiated by the addition of ^14^C-radiolabeled glucose (Moravek Biochemicals) to 50 μL RSO vesicles in 100 mM KPi buffer pH 7.5, at O.D._600nm_ of 2.0. After one minute, the transport was stopped and the radioactivity was measured as described above (see ‘Entrance counter-flow transport assay for human GLUTs’). When inhibitors were used, they were incubated with RSO vesicles 1 minute before transport initiation.

## Additional Information

**How to cite this article**: George Thompson, A. M. *et al*. Discovery of a specific inhibitor of human GLUT5 by virtual screening and *in vitro* transport evaluation. *Sci. Rep*. **6**, 24240; doi: 10.1038/srep24240 (2016).

## Supplementary Material

Supplementary Information

## References

[b1] Vos M. B., Kimmons J. E., Gillespie C., Welsh J. & Blanck H. M. Dietary fructose consumption among US children and adults: the Third National Health and Nutrition Examination Survey. Medscape J. Med. 10, 160 (2008). 18769702 PMC2525476

[b2] Tappy L. & Lê K.-A. Metabolic effects of fructose and the worldwide increase in obesity. Physiol. Rev. 90, 23–46 (2010). 20086073 10.1152/physrev.00019.2009

[b3] Hwang I. S., Ho H., Hoffman B. B. & Reaven G. M. Fructose-induced insulin resistance and hypertension in rats. Hypertension 10, 512–516 (1987). 3311990 10.1161/01.hyp.10.5.512

[b4] Bremer A. A., Mietus-Snyder M. & Lustig R. H. Toward a unifying hypothesis of metabolic syndrome. Pediatrics 129, 557–570 (2012). 22351884 10.1542/peds.2011-2912 PMC3289531

[b5] Burant C. F., Takeda J., Brot-Laroche E., Bell G. I. & Davidson N. O. Fructose transporter in human spermatozoa and small intestine is GLUT5. J. Biol. Chem. 267, 14523–14526 (1992). 1634504

[b6] Thorens B. & Mueckler M. Glucose transporters in the 21st Century. Am. J. Physiol. Endocrinol. Metab. 298, E141–145 (2010). 20009031 10.1152/ajpendo.00712.2009 PMC2822486

[b7] Uldry M. & Thorens B. The SLC2 family of facilitated hexose and polyol transporters. Pflüg. Arch. Eur. J. Physiol. 447, 480–489 (2004). 10.1007/s00424-003-1085-0 12750891

[b8] Douard V. & Ferraris R. P. Regulation of the fructose transporter GLUT5 in health and disease. Am. J. Physiol. - Endocrinol. Metab. 295, E227–E237 (2008). 18398011 10.1152/ajpendo.90245.2008 PMC2652499

[b9] Godoy A. . Differential subcellular distribution of glucose transporters GLUT1–6 and GLUT9 in human cancer: Ultrastructural localization of GLUT1 and GLUT5 in breast tumor tissues. J. Cell. Physiol. 207, 614–627 (2006). 16523487 10.1002/jcp.20606

[b10] Zamora-León S. P. . Expression of the fructose transporter GLUT5 in human breast cancer. Proc. Natl. Acad. Sci. 93, 1847–1852 (1996). 8700847 10.1073/pnas.93.5.1847 PMC39870

[b11] Monzavi-Karbassi B. . Fructose as a carbon source induces an aggressive phenotype in MDA-MB-468 breast tumor cells. Int. J. Oncol. 37, 615–622 (2010). 20664930 10.3892/ijo_00000710 PMC3267577

[b12] Chan K. K., Chan J. Y. W., Chung K. K. W. & Fung K.-P. Inhibition of cell proliferation in human breast tumor cells by antisense oligonucleotides against facilitative glucose transporter 5. J. Cell. Biochem. 93, 1134–1142 (2004). 15449313 10.1002/jcb.20270

[b13] Liu H. . Fructose induces transketolase flux to promote pancreatic cancer growth. Cancer Res. 70, 6368–6376 (2010). 20647326 10.1158/0008-5472.CAN-09-4615

[b14] Soueidan O.-M. . New fluorinated fructose analogs as selective probes of the hexose transporter protein GLUT5. Org. Biomol. Chem. 13, 6511–6521 (2015). 25975431 10.1039/c5ob00314h

[b15] David E. S., Cingari D. S. & Ferraris R. P. Dietary Induction of Intestinal Fructose Absorption in Weaning Rats. Pediatr. Res. 37, 777–782 (1995). 7651763 10.1203/00006450-199506000-00017

[b16] Stuart C. A., Howell M. E. A. & Yin D. Overexpression of GLUT5 in diabetic muscle is reversed by pioglitazone. Diabetes Care 30, 925–931 (2007). 17251278 10.2337/dc06-1788

[b17] George Thompson A. M., Iancu C. V., Nguyen T. T. H., Kim D. & Choe J. Inhibition of human GLUT1 and GLUT5 by plant carbohydrate products; insights into transport specificity. Sci. Rep. 5, 12804 (2015). 26306809 10.1038/srep12804 PMC4549712

[b18] Inukai K. . Characterization of rat GLUT5 and functional analysis of chimeric proteins of GLUT1 glucose transporter and GLUT5 fructose transporter. Endocrinology 136, 4850–4857 (1995). 7588216 10.1210/endo.136.11.7588216

[b19] Gaster M., Staehr P., Beck-Nielsen H., Schrøder H. D. & Handberg A. GLUT4 Is Reduced in Slow Muscle Fibers of Type 2 Diabetic Patients Is Insulin Resistance in Type 2 Diabetes a Slow, Type 1 Fiber Disease? Diabetes 50, 1324–1329 (2001). 11375332 10.2337/diabetes.50.6.1324

[b20] Emsley P., Lohkamp B., Scott W. G. & Cowtan K. Features and development of Coot. Acta Crystallogr. D Biol. Crystallogr. 66, 486–501 (2010). 20383002 10.1107/S0907444910007493 PMC2852313

[b21] Iancu C. V., Zamoon J., Woo S., Aleshin A. & Choe J. Crystal structure of a glucose/H+ symporter and its mechanism of action. Proc. Natl. Acad. Sci. USA 110, 17862–17867 (2013). 24127585 10.1073/pnas.1311485110 PMC3816430

[b22] Deng D. . Crystal structure of the human glucose transporter GLUT1. Nature 510, 121–125 (2014). 24847886 10.1038/nature13306

[b23] Nomura N. . Structure and mechanism of the mammalian fructose transporter GLUT5. Nature 526, 397–401 (2015). 26416735 10.1038/nature14909 PMC4618315

[b24] Krissinel E. & Henrick K. Secondary-structure matching (SSM), a new tool for fast protein structure alignment in three dimensions. Acta Crystallogr. D Biol. Crystallogr. 60, 2256–2268 (2004). 15572779 10.1107/S0907444904026460

[b25] Corpe C. P. . The regulation of GLUT5 and GLUT2 activity in the adaptation of intestinal brush-border fructose transport in diabetes. Pflüg. Arch. Eur. J. Physiol. 432, 192–201 (1996). 10.1007/s004240050124 8662294

[b26] Concha I. I. . Human Erythrocytes Express GLUT5 and Transport Fructose. Blood 89, 7 (1997). 9166863

[b27] Colville C. A., Seatter M. J., Jess T. J., Gould G. W. & Thomas H. M. Kinetic analysis of the liver-type (GLUT2) and brain-type (GLUT3) glucose transporters in Xenopus oocytes: substrate specificities and effects of transport inhibitors. Biochem. J. 290 (Pt 3), 701–706 (1993). 8457197 10.1042/bj2900701 PMC1132337

[b28] Sun L. . Crystal structure of a bacterial homologue of glucose transporters GLUT1-4. Nature 490, 361–366 (2012). 23075985 10.1038/nature11524

[b29] Deng D. . Molecular basis of ligand recognition and transport by glucose transporters. Nature, doi: 10.1038/nature14655 (2015). 26176916

[b30] Hruz P. W. & Mueckler M. M. Cysteine-scanning mutagenesis of transmembrane segment 7 of the GLUT1 glucose transporter. J. Biol. Chem. 274, 36176–36180 (1999). 10593902 10.1074/jbc.274.51.36176

[b31] Bologa C. G. . Virtual and biomolecular screening converge on a selective agonist for GPR30. Nat. Chem. Biol. 2, 207–212 (2006). 16520733 10.1038/nchembio775

[b32] Dennis M. K. . *In vivo* effects of a GPR30 antagonist. Nat. Chem. Biol. 5, 421–427 (2009). 19430488 10.1038/nchembio.168 PMC2864230

[b33] Strouse J. J. . A selective ATP-binding cassette subfamily G member 2 efflux inhibitor revealed via high-throughput flow cytometry. J. Biomol. Screen. 18, 26–38 (2013). 22923785 10.1177/1087057112456875 PMC3623016

[b34] Williamson E. A. . Targeting the transposase domain of the DNA repair component Metnase to enhance chemotherapy. Cancer Res. 72, 6200–6208 (2012). 23090115 10.1158/0008-5472.CAN-12-0313 PMC3972061

[b35] Bologa C. G. & Oprea T. I. Compound collection preparation for virtual screening. Methods Mol. Biol. Clifton NJ 910, 125–143 (2012). 10.1007/978-1-61779-965-5_7 22821595

[b36] McGann M. FRED pose prediction and virtual screening accuracy. J. Chem. Inf. Model. 51, 578–596 (2011). 21323318 10.1021/ci100436p

[b37] Rogers D. & Hahn M. Extended-Connectivity Fingerprints. J. Chem. Inf. Model. 50, 742–754 (2010). 20426451 10.1021/ci100050t

[b38] Hawkins P. C. D., Skillman A. G., Warren G. L., Ellingson B. A. & Stahl M. T. Conformer Generation with OMEGA: Algorithm and Validation Using High Quality Structures from the Protein Databank and Cambridge Structural Database. J. Chem. Inf. Model. 50, 572–584 (2010). 20235588 10.1021/ci100031x PMC2859685

[b39] Halgren T. A. Merck molecular force field. I. Basis, form, scope, parameterization, and performance of MMFF94. J. Comput. Chem. 17, 490–519 (1996).

[b40] Hawkins P. C. D., Skillman A. G. & Nicholls A. Comparison of shape-matching and docking as virtual screening tools. J. Med. Chem. 50, 74–82 (2007). 17201411 10.1021/jm0603365

[b41] McQuade D. T., Plutschack M. B. & Seeberger P. H. Passive fructose transporters in disease: a molecular overview of their structural specificity. Org. Biomol. Chem. 11, 4909–4920 (2013). 23784005 10.1039/c3ob40805a

[b42] Braman J., Papworth C. & Greener A. Site-directed mutagenesis using double-stranded plasmid DNA templates. Methods Mol. Biol. Clifton NJ 57, 31–44 (1996). 10.1385/0-89603-332-5:31 8849992

[b43] Geertsma E. R., Nik Mahmood N. a. B., Schuurman-Wolters G. K. & Poolman B. Membrane reconstitution of ABC transporters and assays of translocator function. Nat. Protoc. 3, 256–266 (2008). 18274528 10.1038/nprot.2007.519

[b44] Kaback H. R. Bacterial membranes. Methods Enzymol. 22, 99–120 (1971).

[b45] Short S. A., Kaback H. R. & Kohn L. D. Localization of D-lactate dehydrogenase in native and reconstituted Escherichia coli membrane vesicles. J. Biol. Chem. 250, 4291–4296 (1975). 1092688

[b46] Kraulis P. J. MOLSCRIPT: a program to produce both detailed and schematic plots of protein structures. J. Appl. Crystallogr. 24, 946–950 (1991).

[b47] Merritt E. A. & Bacon D. J. Raster3D: photorealistic molecular graphics. Methods Enzymol. 277, 505–524 (1997). 18488322 10.1016/s0076-6879(97)77028-9

[b48] Larkin M. A. . Clustal W and Clustal X version 2.0. Bioinforma. Oxf. Engl. 23, 2947–2948 (2007). 10.1093/bioinformatics/btm404 17846036

