# Supplementary material for: Discovery of a specific inhibitor of human GLUT5 by virtual screening and in vitro transport evaluation
Source: Sci Rep. 2016 Apr 14;6:24240. doi: 10.1038/srep24240 (PMC4831007; doi:10.1038/srep24240)
Supplement: Supplementary Information [file srep24240-s1.pdf]

Supplementary Information

Discovery of a specific inhibitor of human GLUT5 by virtual screening and *in vitro* transport evaluation

Alayna M. George Thompson[1]*, Oleg Ursu[2]*, Petr Babkin[1]*, Cristina V. Iancu[1], Alex Whang[1], Tudor I. Oprea[2] and Jun-yong Choe[1]

Supplementary Figure S1

Supplementary Figure S2

Supplementary Figure S3

Supplementary Figure S4

Supplementary Figure S5

Supplementary Table S1

Supplementary Figure S1: Overlay of GLUT5 homology models based on GlcP$_{Se}$ (4LDS), human GLUT1 (4PYP) and bovine GLUT5 (4YB9) crystal structures.

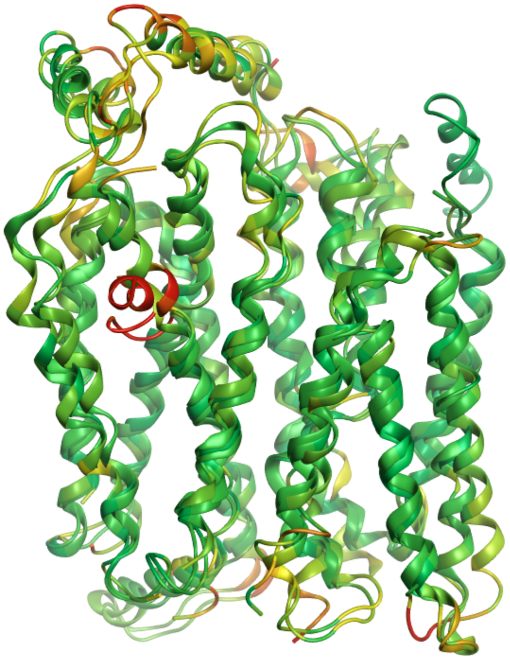

Root mean squared deviation (RMSD) in Cα atoms of GLUT5 homology models are color-coded: green for small deviations (dark green less than 2 Å, light green 2-3 Å), yellow for medium deviations (3-4.5 Å) and red for the largest deviations (up to 10 Å in loop regions). Overall RMSD is ~2.5 Å and 1.5 Å in the transmembrane helix core. Figure was generated in MOE.

Supplementary Figure S2: Glucose uptake into MCF7 cells.

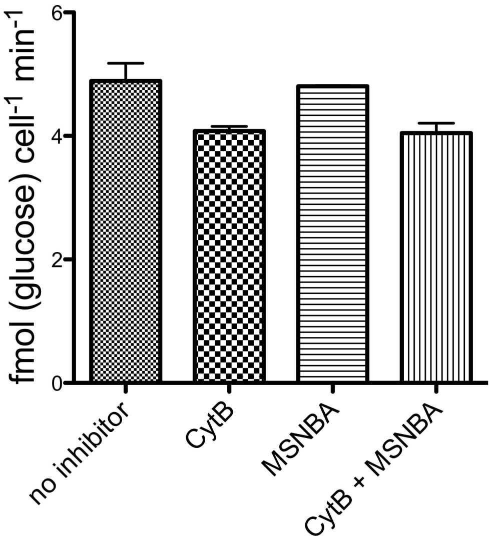

Cytochalasin B (CytB, 50 μM) and MSNBA (60 μM) were pre-incubated with MCF7 cells for five minutes before initiation of uptake by the addition of C^{14}-glucose. Uptake was stopped after thirty minutes and measured as described in Materials and Methods.

Supplementary Figure S3: Time course of fructose uptake in MCF7 cells.

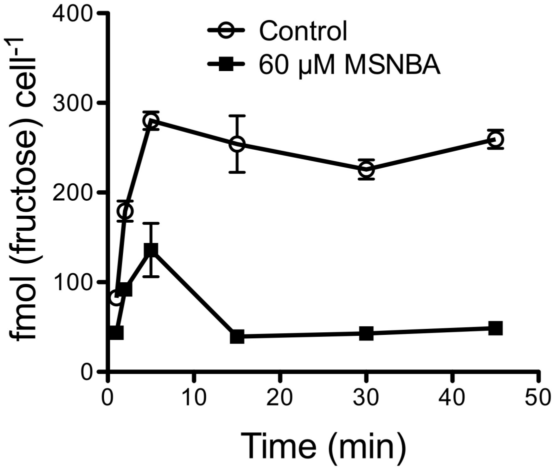

Fructose uptake into MCF7 cells over time with 50 μM cytochalasin B in all conditions in the absence (Control – empty circles) or presence of 60 μM MSNBA (60 μM MSNBA – filled squares). Cells were pre-incubated for five minutes with inhibitors before initiation of uptake by the addition of C^{14}-fructose.

Supplementary Figure S4: Close-up of interactions between MSNBA and side chains of GLUT5.

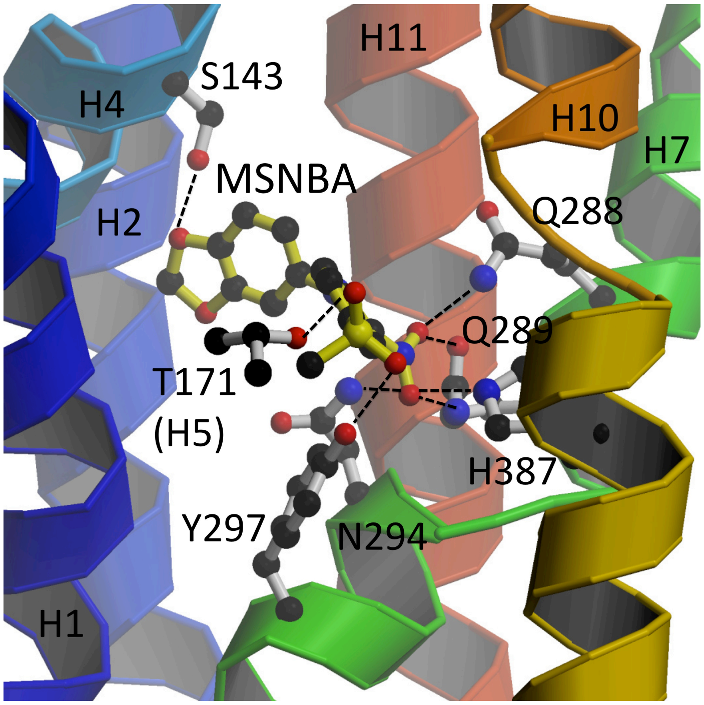

Interacting residues come from helices 4 (S143), 5 (T171), 7 (Q288, Q289, N294 and Y297), and 10 (H387). Helix 5 was omitted for clarity. Figure was drawn with Molscript[46] and raster3D[47].

Supplementary Figure S5: Michaelis-Menten plot for glucose uptake by $GlcP_{Se,F348H}$, in RSO vesicles.

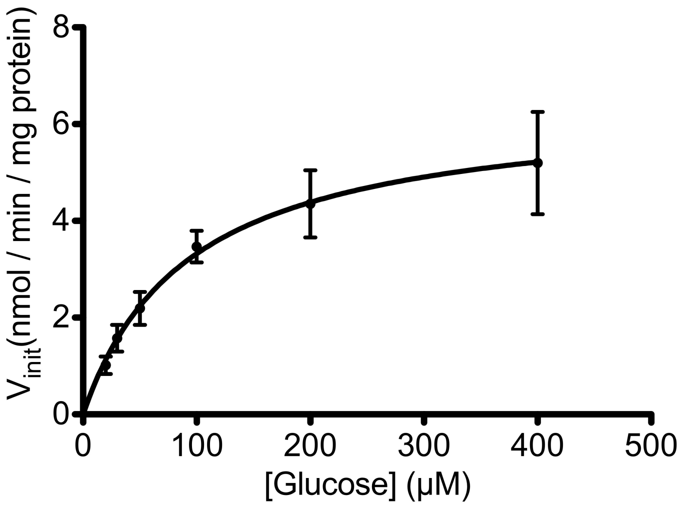

The experiments were conducted in JM1100 *E. coli* cells expressing $GlcP_{Se,F348H}$. Assay was performed at different glucose concentrations and stopped one minute after addition of C^{14}-glucose. Transport kinetic parameters were determined with Prism (GraphPad Software): K_M = 94 ± 19 µM, V_{max} = 6.4 ± 0.5 nmol/min/mg. Error bar is standard deviation from 3 different measurements.

Supplementary Table S1. Compounds tested for GLUT5 transport inhibition in proteoliposomes by entrance counter-flow assay. MSNBA is in bold and red characters.

SMILES	Supplier	MW	CHEM_NAME
[N+](=O)([O-])c1c(ccc(c1)C)NC2OC(C(C2O)O)CO	ChemBridge	284.27	N-(4-methyl-2-nitrophenyl)pentofuranosylamine
[N+](=O)([O-])c1c(cccc1)NC2OC(C(C2O)O)CO	ChemBridge	270.24	N-(2-nitrophenyl)pentofuranosylamine
[N+](=O)([O-])c1c(ccc(c1)C#N)NCc2cc(c(cc2)O)O	Enamine	285.26	4-[(3,4-dihydroxybenzyl)amino]-3-nitrobenzonitrile
[N+](=O)([O-])c1c(ccc(c1)[N+](=O)[O-])NCCCCO	ChemDiv	255.23	4-(2,4-dinitroanilino)-1-butanol
N(CC2OC(C(C2O)O)CO)c1ccc(cc1)C	Labotest	253.3	2,5-anhydro-1-deoxy-1-(4-toluidino)hexitol
[S](=O)(=O)(C)c1cc(c(cc1)NCc2c(ccc(c2)OC)OC)[N+](=O)[O-]	Enamine	366.39	N-(2,5-dimethoxybenzyl)-N-[4-(methylsulfonyl)-2-nitrophenyl]amine
[S](=O)(=O)(C)c1cc(c(cc1)NCC2OCCC2)[N+](=O)[O-]	Enamine	300.34	4-(methylsulfonyl)-2-nitro-N-(tetrahydro-2-furanylmethyl)aniline
[N+](=O)([O-])c1c(ccc(c1)[N+](=O)[O-])NCCNCCO	Vitas-M	270.24	2-{[2-(2,4-dinitroanilino)ethyl]amino}ethanol
[N+](=O)([O-])c1ccc(cc1)NC2OC(C(C2O)O)CO	ChemBridge	270.24	N-(4-nitrophenyl)pentofuranosylamine
Clc1c(ccc(c1)[N+](=O)[O-])NCc2cc(c(cc2)OC)O	Enamine	308.72	5-[(2-chloro-4-nitroanilino)methyl]-2-methoxyphenol
[n]2(ncnc2)C1OC(C(C1O)O)CO	AMS Private	201.18	2-(hydroxymethyl)-5-(1H-1,2,4-triazol-1-yl)tetrahydrofuran-3,4-diol
[S](=O)(=O)(C)c1cc(c(cc1)NCCc2nc(n[o]2)N)[N+](=O)[O-]	Enamine	327.32	N-[2-(3-amino-1,2,4-oxadiazol-5-yl)ethyl]-N-[4-(methylsulfonyl)-2-nitrophenyl]amine
[N+](=O)([O-])c1cc(c(cc1)NCc2ccc(cc2)N)C(=O)OC	Key Organics / BIONET	301.3	methyl 2-[(4-aminobenzyl)amino]-5-nitrobenzoate
[N+](=O)([O-])c1c(ccc(c1)C(=O)C)NCc2cc(c(c(c2)C)O)C	Enamine	314.34	1-{4-[(4-hydroxy-3,5-dimethylbenzyl)amino]-3-nitrophenyl}ethanone
[N+](=O)([O-])c1cc(c(cc1)NCc2[o]ccc2)C(=O)O	Maybridge Ltd	262.22	2-[(2-furylmethyl)amino]-5-nitrobenzoic acid
[N+](=O)([O-])c1c(ccc(c1)C(=O)C)NCc2c(nc(cc2C)C)O	Enamine	315.33	1-(4-{[(2-hydroxy-4,6-dimethyl-3-pyridinyl)methyl]amino}-3-nitrophenyl)ethanone
[N+](=O)([O-])c1cc(c(cc1)NCC2C(CCC2)O)C(=O)NC	Enamine	293.32	2-{[(2-hydroxycyclopentyl)methyl]amino}-N-methyl-5-nitrobenzamide
[S](=O)(=O)(C)c1cc(c(cc1)NCc2[o]ccc2)[N+](=O)[O-]	Enamine	296.3	N-(2-furylmethyl)-4-(methylsulfonyl)-2-nitroaniline
[S](=O)(=O)(N)c1cc(c(cc1)NCC2OCCC2)[N+](=O)[O-]	Enamine	301.32	3-nitro-4-[(tetrahydro-2-furanylmethyl)amino]benzenesulfonamide
[N+](=O)([O-])c1c(ccc(c1)C(=O)C)NCc2c(ccc(c2)OC)OC	Enamine	330.34	1-{4-[(2,5-dimethoxybenzyl)amino]-3-nitrophenyl}ethanone
[S](=O)(=O)(N)c1cc(c(cc1)NCCc2c([nH]nc2O)C)[N+](=O)[O-]	Enamine	341.35	4-{[2-(3-hydroxy-5-methyl-1H-pyrazol-4-yl)ethyl]amino}-3-nitrobenzenesulfonamide
[N+](=O)([O-])c1c(ccc(c1)C(=O)O)NC(C)c2ncccc2	Enamine	287.27	3-nitro-4-{[1-(2-pyridinyl)ethyl]amino}benzoic acid
S([C@H]2OC(C(C2O)O)[C@@H](O)CO)c1ccccc1	Sigma-Aldrich	272.32	phenyl 1-thio-beta-L-erythro-hexofuranoside
Fc1c(ccc(c1)[N+](=O)[O-])NCc2cc(c(cc2)O)OC	Enamine	292.27	4-[(2-fluoro-4-nitroanilino)methyl]-2-methoxyphenol
[s]1c(nc(c1)CNc2c(cc(cc2)C(=O)C)[N+](=O)[O-])NC	Enamine	306.35	1-[4-({[2-(methylamino)-1,3-thiazol-4-yl]methyl}amino)-3-nitrophenyl]ethanone

[N+](=O)([O-])c1c(ccc(c1)C(=O)C)Nc2cc(c(cc2)OC)O	Enamine	302.29	1-[4-(3-hydroxy-4-methoxyanilino)-3-nitrophenyl]ethanone
[N+](=O)([O-])c1c(ccc(c1)C(=O)O)NCc2ccccc2	ChemDiv	272.26	4-(benzylamino)-3-nitrobenzoic acid
[N+](=O)([O-])c1c(ccc(c1)c3n[o]c(n3)C)NCC2OCCC2	Vitas-M	304.31	N-[4-(5-methyl-1,2,4-oxadiazol-3-yl)-2-nitrophenyl]-N-(tetrahydro-2-furanylmethyl)amine
[N+](=O)([O-])c1cc(c(cc1)NCc2c(nc(cc2C)C)O)COC	Enamine	317.34	
[Na+].[S](=O)(=O)([O-])CCNc1c(cc(cc1)[N+](=O)[O-])[N+](=O)[O-]	Sigma-Aldrich	313.22	sodium 2-(2,4-dinitroanilino)ethanesulfonate
[N+](=O)([O-])c1cc(c(cc1)NCc2nc[o]c2C)C(=O)OC	Enamine	291.26	
[S](=O)(=O)(C)c1cc(c(cc1)Nc2cc(c(cc2)OC)OC)[N+](=O)[O-]	Enamine	352.37	N-(3,4-dimethoxyphenyl)-4-(methylsulfonyl)-2-nitroaniline
Clc1cc(c(cc1)NC2OC(C(C(C2O)O)O)C)[N+](=O)[O-]	ChemBridge	318.71	N-(4-chloro-2-nitrophenyl)-6-deoxyhexopyranosylamine
[N+](=O)([O-])c1cc(c(cc1)NCc2c(c(ccc2)OC)O)OC	Sigma-Aldrich	304.3	2-methoxy-6-[(2-methoxy-4-nitroanilino)methyl]phenol
O1C(C(C(C1CO)O)O)OC(=O)c2ccccc2	Sigma-Aldrich	254.24	1-O-benzoyl-D-ribofuranose
[N+](=O)([O-])c1c(ccc(c1)C(=O)C)NC2CCC(CC2)O	Enamine	278.31	1-{4-[(4-hydroxycyclohexyl)amino]-3-nitrophenyl}ethanone
FC(F)(F)c1cc(c(cc1)NCc2cc(c(cc2)OC)OC)[N+](=O)[O-]	ChemBridge	356.3	N-(3,4-dimethoxybenzyl)-2-nitro-4-(trifluoromethyl)aniline
[N+](=O)([O-])c1c(ccc(c1)C(=O)C)NCC(CO)Cc2[o]ccc2	Enamine	318.33	1-(4-{[3-(2-furyl)-2-(hydroxymethyl)propyl]amino}-3-nitrophenyl)ethanone
[S](=O)(=O)(C)c1cc(c(cc1)NCc2cc(ncc2)OC)[N+](=O)[O-]	Enamine	337.36	N-[(2-methoxy-4-pyridinyl)methyl]-N-[4-(methylsulfonyl)-2-nitrophenyl]amine
[S](=O)(=O)(N)c1cc(c(cc1)NCC(=O)N2CCC(CC2)O)[N+](=O)[O-]	Enamine	358.38	4-{[2-(4-hydroxy-1-piperidinyl)-2-oxoethyl]amino}-3-nitrobenzenesulfonamide
FC(F)(F)c1c(cc(c(c1)[N+](=O)[O-])NCc2nc(c(cc2)C(O)C)C)Cl	Enamine	389.76	
Fc1c(ccc(c1)C(Nc2c(cc(cc2)[S](=O)(=O)C)[N+](=O)[O-])C)OC	Enamine	368.39	N-[1-(3-fluoro-4-methoxyphenyl)ethyl]-4-(methylsulfonyl)-2-nitroaniline
FC(F)(F)C(O)CCNc1c(cc(cc1)[N+](=O)[O-])C(=O)OC	Enamine	322.24	methyl 5-nitro-2-[(4,4,4-trifluoro-3-hydroxybutyl)amino]benzoate
[S](=O)(=O)(C)c1c(ccc(c1)[S](=O)(=O)C)NCCOCCO	Enamine	337.42	2-{2-[2,4-bis(methylsulfonyl)anilino]ethoxy}ethanol
[N+](=O)([O-])c1c(ccc(c1)C(=O)N)NCc2c(cccc2)OC	Enamine	301.3	4-[(2-methoxybenzyl)amino]-3-nitrobenzamide
[N+](=O)([O-])c1c(ccc(c1)OC)NC(=O)N2CC(CCC2)O	Sigma-Aldrich	295.3	3-hydroxy-N-(4-methoxy-2-nitrophenyl)-1-piperidinecarboxamide
[S](=O)(=O)(NC)c1cc(c(cc1)NC2CCC(CC2)O)[N+](=O)[O-]	Enamine	329.38	4-[(4-hydroxycyclohexyl)amino]-N-methyl-3-nitrobenzenesulfonamide
[S](=O)(=O)(C)c1cc(c(cc1)NCCc2ncccc2)[N+](=O)[O-]	Enamine	321.36	4-(methylsulfonyl)-2-nitro-N-[2-(2-pyridinyl)ethyl]aniline
[N+](=O)([O-])c1c(ccc(c1)[N+](=O)[O-])NCCCCCC(=O)O	Sigma-Aldrich	297.27	6-(2,4-dinitroanilino)hexanoic acid
FC(F)[S](=O)(=O)c1cc(c(cc1)NCc2n[o]c(c2)C)[N+](=O)[O-]	Enamine	347.3	4-[(difluoromethyl)sulfonyl]-N-[(5-methyl-3-isoxazolyl)methyl]-2-nitroaniline
[S](=O)(=O)(N)c1cc(c(cc1)NCC2Oc3c(cccc3)OC2)[N+](=O)[O-]	Enamine	365.37	4-[(2,3-dihydro-1,4-benzodioxin-2-ylmethyl)amino]-3-nitrobenzenesulfonamide
[N+](=O)([O-])c1c(ccc(c1)C(=O)C)NCC2CN(CC2)C	Enamine	277.32	1-(4-{[(1-methyl-3-pyrrolidinyl)methyl]amino}-3-nitrophenyl)ethanone
[N+](=O)([O-])c1c(ccc(c1)[N+](=O)[O-])NCCC(=O)O	Sigma-Aldrich	255.19	3-(2,4-dinitroanilino)propanoic acid
[N+](=O)([O-])c1cc(c(cc1)NCCc2[nH]nc(c2)O)C(=O)OC	Enamine	306.28	

[N+](=O)([O-])c1cc2c(ncnc2O)cc1NCCc3cc(ccc3)O	Enamine	326.31	7-{[2-(3-hydroxyphenyl)ethyl]amino}-6-nitro-4-quinazolinol
[N+](=O)([O-])c1c(ccc(c1)C(=O)O)Nc2ccc(cc2)OC	ChemDiv	288.26	4-(4-methoxyanilino)-3-nitrobenzoic acid
[N+](=O)([O-])c1c(ccc(c1)C(=O)N)NCC(CO)Cc2[o]ccc2	Enamine	319.32	4-{[3-(2-furyl)-2-(hydroxymethyl)propyl]amino}-3-nitrobenzamide
FC(F)[S](=O)(=O)c1cc(c(cc1)NCc2nc[s]c2)[N+](=O)[O-]	Enamine	349.34	4-[(difluoromethyl)sulfonyl]-2-nitro-N-(1,3-thiazol-4-ylmethyl)aniline
[N+](=O)([O-])c1cc(c(cc1)NCC2OCCOC2)C(=O)OC	Enamine	296.28	methyl 2-[(1,4-dioxan-2-ylmethyl)amino]-5-nitrobenzoate
[N+](=O)([O-])c1cc(c(cc1)NC2CCC(CC2)O)C(=O)OC	Enamine	294.31	methyl 2-[(4-hydroxycyclohexyl)amino]-5-nitrobenzoate
[S](=O)(=O)(C)c1cc(c(cc1)Nc2cnc(cc2)N3CCCC3)[N+](=O)[O-]	Enamine	362.41	N-[4-(methylsulfonyl)-2-nitrophenyl]-6-(1-pyrrolidinyl)-3-pyridinamine
[N+](=O)([O-])c1cc(c(cc1)NCc2c(ccc(c2)OC)OC)C	Vitas-M	302.33	N-(2,5-dimethoxybenzyl)-2-methyl-4-nitroaniline
[N+](=O)([O-])c1c(ccc(c1)C#N)NCc2c(ccc(c2)OC)OC	Enamine	313.31	4-[(2,5-dimethoxybenzyl)amino]-3-nitrobenzonitrile
[S](=O)(=O)(NC)c1cc(c(cc1)NCC2(CCC2)O)[N+](=O)[O-]	Enamine	315.35	4-{[(1-hydroxycyclobutyl)methyl]amino}-N-methyl-3-nitrobenzenesulfonamide
[N+](=O)([O-])c1c(ccc(c1)C(=O)C)NC(C)c2ncccc2	Enamine	285.3	1-(3-nitro-4-{[1-(2-pyridinyl)ethyl]amino}phenyl)ethanone
[N+](=O)([O-])c1cc2c(ncnc2O)cc1NCC3OCCC3	Enamine	290.28	6-nitro-7-[(tetrahydro-2-furanylmethyl)amino]-4-quinazolinol
[N+](=O)([O-])c1c(ccc(c1)C(=O)OC)NC(C)c2ncccc2	Enamine	301.3	methyl 3-nitro-4-{[1-(2-pyridinyl)ethyl]amino}benzoate
FC(F)[S](=O)(=O)c1cc(c(cc1)NCc2n[nH]c(n2)C)[N+](=O)[O-]	Enamine	347.3	4-[(difluoromethyl)sulfonyl]-N-[(5-methyl-1H-1,2,4-triazol-3-yl)methyl]-2-nitroaniline
[S](=O)(=O)(C)c1cc(c(cc1)NCC2CN(CC2)CCOC)[N+](=O)[O-]	Enamine	357.43	N-{[1-(2-methoxyethyl)-3-pyrrolidinyl]methyl}-N-[4-(methylsulfonyl)-2-nitrophenyl]amine
[N+](=O)([O-])c1c(ccc(c1)C(=O)O)NCCc2c3c([nH]c2)cccc3	Vitas-M	325.32	4-{[2-(1H-indol-3-yl)ethyl]amino}-3-nitrobenzoic acid
[N+](=O)([O-])c1c(ccc(c1)C(=O)O)NCCc2cc(c(cc2)O)O	Vitas-M	318.29	4-(3,4-dihydroxyphenethylamino)-3-nitrobenzoic acid
[S](=O)(=O)(O)c1c(ccc(c1)[N+](=O)[O-])Nc2ccc(cc2)N	ChemBridge	309.3	2-(4-aminoanilino)-5-nitrobenzenesulfonic acid
[N+](=O)([O-])c1cc(c(cc1)NCC2OCCC2)C#N	Enamine	247.25	5-nitro-2-[(tetrahydro-2-furanylmethyl)amino]benzonitrile
[N+](=O)([O-])c1c(ccc(c1)[N+](=O)[O-])NCCCN(C)C	Vitas-M	304.73	N~1~-(2,4-dinitrophenyl)-N~3~,N~3~-dimethyl-1,3-propanediamine
[S](=O)(=O)(C)c1cc(c(cc1)NCC2(CC(CCC2)C)O)[N+](=O)[O-]	Enamine	342.42	3-methyl-1-{[4-(methylsulfonyl)-2-nitroanilino]methyl}cyclohexanol
[N+](=O)([O-])c1c(ccc(c1)c3n[o]c(n3)C)NCc2[o]ccc2	Vitas-M	300.27	N-(2-furylmethyl)-4-(5-methyl-1,2,4-oxadiazol-3-yl)-2-nitroaniline
[S](=O)(=O)(C)c1cc(c(cc1)NCc2[n]3c(nn2)CCC3)[N+](=O)[O-]	Enamine	337.36	N-(6,7-dihydro-5H-pyrrolo[2,1-c][1,2,4]triazol-3-ylmethyl)-N-[4-(methylsulfonyl)-2-nitrophenyl]amine
Clc1c(ccc(c1)[N+](=O)[O-])NCc2cc(c(cc2)OC)OC	Enamine	322.75	2-chloro-N-(3,4-dimethoxybenzyl)-4-nitroaniline
Fc1c(ccc(c1)[N+](=O)[O-])NCc2ccc(cc2)C(O)C	Enamine	290.29	1-{4-[(2-fluoro-4-nitroanilino)methyl]phenyl}ethanol
[N+](=O)([O-])c1ccc(cc1)O[C@@H]2O[C@@H]([C@H]([C@@H]([C@H]2O)O)O)CO	Sigma-Aldrich	301.25	4-nitrophenyl beta-D-glucopyranoside
[N+](=O)([O-])c1c(ccc(c1)C(=O)C)NCCc2c(ccc(c2)C)OC	Enamine	328.37	1-(4-{[2-(2-methoxy-5-methylphenyl)ethyl]amino}-3-nitrophenyl)ethanone
Clc1c(ccc(c1)[N+](=O)[O-])NCc2c(ccc(c2)OC)OC	Enamine	322.75	2-chloro-N-(2,5-dimethoxybenzyl)-4-nitroaniline

[N+](=O)([O-])c1c(ccc(c1)OC)NC(=O)N2CCC(CC2)O	Enamine	295.3	4-hydroxy-N-(4-methoxy-2-nitrophenyl)-1-piperidinecarboxamide
[S](=O)(=O)(NC)c1cc(c(cc1)Nc2cnc(cc2)N(C)C)[N+](=O)[O-]	Enamine	351.39	4-{[6-(dimethylamino)-3-pyridinyl]amino}-N-methyl-3-nitrobenzenesulfonamide
[S]1(=O)(=O)N=C(c3c1cc(cc3)[N+](=O)[O-])Nc2cc(ccc2)N	Vitas-M	318.31	
[S](=O)(=O)(C)c1cc(c(cc1)NC(C)c2cc3c(cc2)OCO3)[N+](=O)[O-]	Enamine	364.38	N-[1-(1,3-benzodioxol-5-yl)ethyl]-4-(methylsulfonyl)-2-nitroaniline
[N+](=O)([O-])c1cc(c(cc1)NCc2nc(n[o]2)C(OCC)C)C#N	Enamine	317.3	2-({[3-(1-ethoxyethyl)-1,2,4-oxadiazol-5-yl]methyl}amino)-5-nitrobenzonitrile
[N+](=O)([O-])c1c(ccc(c1)C#N)NC2CCC(CC2)O	Enamine	261.28	4-[(4-hydroxycyclohexyl)amino]-3-nitrobenzonitrile
[N+](=O)([O-])c1c(ccc(c1)[N+](=O)[O-])NC(C(O)C)C(=O)O	Sigma-Aldrich	285.21	2-(2,4-dinitroanilino)-3-hydroxybutanoic acid
[S](=O)(=O)(C)c1cc(c(cc1)NCC2N(CCC2)CC)[N+](=O)[O-]	Enamine	327.4	N-[(1-ethyl-2-pyrrolidinyl)methyl]-4-(methylsulfonyl)-2-nitroaniline
[N+](=O)([O-])c1ccc(cc1)N[C@@H]2O[C@@H]([C@H]([C@@H]([C@@H]2O)O)O)CO	Vitas-M	300.27	N-(4-nitrophenyl)-beta-D-mannopyranosylamine
Brc1c(cc(c(c1)NCC(O)C(O)C(O)CO)[N+](=O)[O-])C	Sigma-Aldrich	365.18	1-(5-bromo-4-methyl-2-nitroanilino)-1-deoxy-D-ribitol
[N+](=O)([O-])c1cc(c(cc1)NCc2ccc(cc2)C(=O)OC)C(=O)OC	Enamine	344.32	methyl 2-{[4-(methoxycarbonyl)benzyl]amino}-5-nitrobenzoate
[s]1c(nc(c1)CNc2c(cc(cc2)C(=O)NC)[N+](=O)[O-])NC	Enamine	321.36	N-methyl-4-({[2-(methylamino)-1,3-thiazol-4-yl]methyl}amino)-3-nitrobenzamide
[S](=O)(=O)(C)c1cc(c(cc1)Nc2cc(cc(c2)OC)OC)[N+](=O)[O-]	Enamine	352.37	N-(3,5-dimethoxyphenyl)-4-(methylsulfonyl)-2-nitroaniline
[N+](=O)([O-])c1c(ccc(c1)C(=O)C)NCCc2c3c([nH]c2O)cccc3	Enamine	339.35	3-[2-(4-acetyl-2-nitroanilino)ethyl]-1,3-dihydro-2H-indol-2-one
[S]1(=O)(=O)N=C(c3c1cc(cc3)[N+](=O)[O-])Nc2c(ccc(c2)OC)OC	Vitas-M	363.35	N-(2,5-dimethoxyphenyl)-6-nitro-1,2-benzisothiazol-3-amine 1,1-dioxide
S1c2[n](cc(n2)CNc3c(cc(cc3)C(=O)OC)[N+](=O)[O-])C=C1	Enamine	332.34	methyl 4-[(imidazo[2,1-b][1,3]thiazol-6-ylmethyl)amino]-3-nitrobenzoate
[S](=O)(=O)(NC)c1cc(c(cc1)NCC2Oc3c(cccc3)OC2)[N+](=O)[O-]	Enamine	379.39	4-[(2,3-dihydro-1,4-benzodioxin-2-ylmethyl)amino]-N-methyl-3-nitrobenzenesulfonamide
[n]2(cnc(c2N)C(=O)N)C1OC(C(C1O)O)CO	AMS Private	258.23	5-amino-1-(3,4-dihydroxy-5-(hydroxymethyl)tetrahydrofuran-2-yl)-1H-imidazole-4-carboxamide
[S]1(=O)(=O)N=C(c3c1cc(cc3)[N+](=O)[O-])Nc2cc(ccc2)C(=O)C	Vitas-M	345.34	1-{3-[(6-nitro-1,1-dioxido-1,2-benzisothiazol-3-yl)amino]phenyl}ethanone
Clc1ccc(cc1)C(O)CNc2c(cc(cc2)C(=O)C)[N+](=O)[O-]	Enamine	334.76	1-(4-{[2-(4-chlorophenyl)-2-hydroxyethyl]amino}-3-nitrophenyl)ethanone
[S](=O)(=O)(C)c1c(ccc(c1)[S](=O)(=O)C)NCC2OCCC2	Enamine	333.43	2,4-bis(methylsulfonyl)-N-(tetrahydro-2-furanylmethyl)aniline
Clc1c(ccc(c1)[N+](=O)[O-])NCc2c(nc(cc2C)C)O	Enamine	307.74	3-[(2-chloro-4-nitroanilino)methyl]-4,6-dimethyl-2-pyridinol
Fc1c(c(cc(c1)C(=O)OC)[N+](=O)[O-])NCC2OCCC2	Enamine	298.27	methyl 3-fluoro-5-nitro-4-[(tetrahydro-2-furanylmethyl)amino]benzoate
Fc1c(ccc(c1)CNc2c(cc(cc2)F)[N+](=O)[O-])CO	Enamine	294.26	{2-fluoro-4-[(4-fluoro-2-nitroanilino)methyl]phenyl}methanol
Clc1c(cccc1)CNc2c(cc(cc2)[N+](=O)[O-])C(=O)O	Enamine	306.71	2-[(2-chlorobenzyl)amino]-5-nitrobenzoic acid
[N+](=O)([O-])c1c(ccc(c1)C(=O)OCC)NCc2ccc(cc2)OC	ChemDiv	330.34	ethyl 4-[(4-methoxybenzyl)amino]-3-nitrobenzoate
[N+](=O)([O-])c1c(ccc(c1)C(=O)OC)NCc2nc3[n](c2)C=CC=C3	Enamine	326.31	methyl 4-[(imidazo[1,2-a]pyridin-2-ylmethyl)amino]-3-nitrobenzoate

[N+](=O)([O-])c1cc(c(cc1)NCc2c(cc(cc2)OC)OC)C	Vitas-M	302.33	N-(2,4-dimethoxybenzyl)-2-methyl-4-nitroaniline
Clc1ccc(cc1)CN[C@@H]2OC([C@H](C(C2O)O)O)CO	ChemDiv	303.74	N-(4-chlorobenzyl)-beta-D-glycero-hexopyranosylamine
Fc1ccc(cc1)CNc2c(cc(cc2)C(=O)N)[N+](=O)[O-]	Enamine	289.27	4-[(4-fluorobenzyl)amino]-3-nitrobenzamide
[N+](=O)([O-])c1cc(c(cc1)NCc2ccc(cc2)OC)C#N	Enamine	283.29	2-[(4-methoxybenzyl)amino]-5-nitrobenzonitrile
[N+](=O)([O-])c1c(ccc(c1)C(=O)O)Nc2ccc(cc2)C	Vitas-M	272.26	3-nitro-4-(4-toluidino)benzoic acid
[N+](=O)([O-])c1cc(c(cc1)N\C=C2/CCCC/2=O)OC	Vitas-M	262.27	(2E)-2-[(2-methoxy-4-nitroanilino)methylene]cyclopentanone
FC(F)(F)c1cc(c(cc1)NCc2ccc(cc2)OC)[N+](=O)[O-]	Enamine	326.28	N-(4-methoxybenzyl)-2-nitro-4-(trifluoromethyl)aniline
S(c2nc(cc(n2)O)N)c1c(cc(cc1)C(=O)C)[N+](=O)[O-]	Labotest	306.3	1-{4-[(4-amino-6-hydroxy-2-pyrimidinyl)sulfanyl]-3-nitrophenyl}ethanone
[N+](=O)([O-])c1c(ccc(c1)C(=O)N)NCc2ccc(cc2)C	Enamine	285.3	4-[(4-methylbenzyl)amino]-3-nitrobenzamide
[S](=O)(=O)(C)c1cc(c(cc1)Sc2nc(cc(n2)C)C)[N+](=O)[O-]	Enamine	339.4	4,6-dimethyl-2-{[4-(methylsulfonyl)-2-nitrophenyl]sulfanyl}pyrimidine
**[S](=O)(=O)(C)c1cc(c(cc1)Nc2cc3c(cc2)OCO3)[N+](=O)[O-]**	**Enamine**	**336.33**	**N-[4-(methylsulfonyl)-2-nitrophenyl]-1,3-benzodioxol-5-amine, MSNBA**
[N+](=O)([O-])c1c(ccc(c1)C(=O)O)NCCC2=CCCCC2	ChemDiv	290.32	4-{[2-(1-cyclohexen-1-yl)ethyl]amino}-3-nitrobenzoic acid
[N+](=O)([O-])c1cc(c(cc1)NCc2cnccc2)C(=O)O	ChemBridge	273.25	5-nitro-2-[(3-pyridinylmethyl)amino]benzoic acid
[S](=O)(=O)(N)c1cc(c(cc1)NCc2ccccc2)[N+](=O)[O-]	ChemBridge	307.33	4-(benzylamino)-3-nitrobenzenesulfonamide
[S](=O)(=O)(C)c1cc(c(cc1)NC(C)c2[s]ccc2)[N+](=O)[O-]	Enamine	326.4	4-(methylsulfonyl)-2-nitro-N-[1-(2-thienyl)ethyl]aniline
Clc1ccc(cc1)CNc2c(cc(cc2)C(=O)N)[N+](=O)[O-]	Enamine	305.72	4-[(4-chlorobenzyl)amino]-3-nitrobenzamide
Fc1c(ccc(c1)[N+](=O)[O-])NCc2cc(c(cc2)CO)F	Enamine	294.26	{2-fluoro-4-[(2-fluoro-4-nitroanilino)methyl]phenyl}methanol
[N+](=O)([O-])c1c(ccc(c1)C(=O)C)NCC2CCN(CC2)CC	Enamine	305.38	1-(4-{[(1-ethyl-4-piperidinyl)methyl]amino}-3-nitrophenyl)ethanone
[N+](=O)([O-])c1c(ccc(c1)C(=O)C)NCc2cc(ncc2)OC	Enamine	301.3	1-(4-{[(2-methoxy-4-pyridinyl)methyl]amino}-3-nitrophenyl)ethanone
Fc1c(ccc(c1)CNc2c(cc(cc2)[N+](=O)[O-])Cl)CO	Enamine	310.71	{4-[(2-chloro-4-nitroanilino)methyl]-2-fluorophenyl}methanol
[N+](=O)([O-])c1cc(c(cc1)NCC2Oc3c(cccc3)OC2)C(=O)OC	Enamine	344.32	methyl 2-[(2,3-dihydro-1,4-benzodioxin-2-ylmethyl)amino]-5-nitrobenzoate
Clc1c(c(cc(c1)Cl)C(=O)Nc2c(cc(cc2)[N+](=O)[O-])OC)O	Vitas-M	357.15	3,5-dichloro-2-hydroxy-N-(2-methoxy-4-nitrophenyl)benzamide
[S](=O)(=O)(C)c1cc(c(cc1)NCC2(CCC2)O)[N+](=O)[O-]	Enamine	300.34	1-{[4-(methylsulfonyl)-2-nitroanilino]methyl}cyclobutanol
[N+](=O)([O-])c1cc(c(cc1)N\C=C2\CCCCC\2=O)OC	ChemBridge	276.29	(2Z)-2-[(2-methoxy-4-nitroanilino)methylene]cyclohexanone
Clc1cc(c(cc1)O)C(=O)Nc2c(cc(cc2)OC)[N+](=O)[O-]	Sigma-Aldrich	322.7	5-chloro-2-hydroxy-N-(4-methoxy-2-nitrophenyl)benzamide
[s]1c(nc(c1CC(=O)Nc2c(cc(cc2)OCC)[N+](=O)[O-])O)N	Vitas-M	338.34	N-(4-ethoxy-2-nitrophenyl)-2-(2-imino-4-oxo-1,3-thiazolidin-5-yl)acetamide
[S](=O)(=O)(NC)c1cc(c(cc1)NCc2cc(ncc2)OC)[N+](=O)[O-]	Enamine	352.37	4-{[(2-methoxy-4-pyridinyl)methyl]amino}-N-methyl-3-nitrobenzenesulfonamide
[N+](=O)([O-])c1cc(c(cc1)NCc2cc(ccc2)OCC)C#N	Enamine	297.31	2-[(3-ethoxybenzyl)amino]-5-nitrobenzonitrile
[s]1c(nc(c1CC(=O)Nc2c(cc(cc2)OC)[N+](=O)[O-])O)N	Vitas-M	324.32	2-(2-imino-4-oxo-1,3-thiazolidin-5-yl)-N-(4-methoxy-2-nitrophenyl)acetamide
[N+](=O)([O-])c1c(ccc(c1)C(=O)OCC)NCc2c(cccc2)OC	ChemDiv	330.34	ethyl 4-[(2-methoxybenzyl)amino]-3-nitrobenzoate

FC(F)(F)c1cc(c(cc1)NC2CCC(CC2)O)[N+](=O)[O-]	Enamine	304.27	4-[2-nitro-4-(trifluoromethyl)anilino]cyclohexanol
[N+](=O)([O-])c1cc(c(cc1)NC2OC(C(C2)O)CO)C	ChemBridge	268.27	2-deoxy-N-(2-methyl-4-nitrophenyl)pentofuranosylamine
Fc1c(cccc1)C(O)CNc2c(cc(cc2)C(=O)C)[N+](=O)[O-]	Enamine	318.3	1-(4-{[2-(2-fluorophenyl)-2-hydroxyethyl]amino}-3-nitrophenyl)ethanone
[s]1c(nnc1N)Sc2c(cc(cc2)[N+](=O)[O-])C(=O)O	Labotest	298.3	2-[(5-amino-1,3,4-thiadiazol-2-yl)sulfanyl]-5-nitrobenzoic acid
Clc1c(cccc1)CCNc2c(cc(cc2)C(=O)O)[N+](=O)[O-]	ChemDiv	320.73	4-{[2-(2-chlorophenyl)ethyl]amino}-3-nitrobenzoic acid
[N+](=O)([O-])c1cc(c(cc1)NCc2cnccc2)C(=O)N	Vitas-M	272.26	5-nitro-2-[(3-pyridinylmethyl)amino]benzamide
Fc1ccc(cc1)CNc2c(cc(cc2)C(=O)OCC)[N+](=O)[O-]	ChemDiv	318.3	ethyl 4-[(4-fluorobenzyl)amino]-3-nitrobenzoate
[S](=O)(=O)(C)c1cc(c(cc1)NC(C)c2ccccc2)[N+](=O)[O-]	Vitas-M	320.37	4-(methylsulfonyl)-2-nitro-N-(1-phenylethyl)aniline
[n]2(ncnc2)C1OC(C(C1O)O)COC	AMS Private Supplier 1	215.21	
O1[C@H](C(C(C1CO)O)O)OCc2ccccc2	Sigma-Aldrich	240.26	benzyl beta-D-ribofuranoside
[S](=O)(=O)(C)c1cc(c(cc1)Sc2[s]cc(n2)C)[N+](=O)[O-]	Enamine	330.41	methyl 4-[(4-methyl-1,3-thiazol-2-yl)sulfanyl]-3-nitrophenyl sulfone
Fc1c(ccc(c1)[N+](=O)[O-])NCC2OCCC2	Enamine	240.23	N-(2-fluoro-4-nitrophenyl)-N-(tetrahydro-2-furanylmethyl)amine
[S](=O)(=O)(C)c1c(ccc(c1)[S](=O)(=O)C)NCCC2OCCC2	Enamine	347.46	2,4-bis(methylsulfonyl)-N-(2-tetrahydro-2-furanylethyl)aniline
Clc1c(ccc(c1)[N+](=O)[O-])NCc2ncccc2	Enamine	263.68	2-chloro-4-nitro-N-(2-pyridinylmethyl)aniline
[N+](=O)([O-])c1c(cccc1)O[C@@H]2O[C@@H]([C@H]([C@@H]([C@H]2O)O)O)CO	Sigma-Aldrich	301.25	2-nitrophenyl beta-D-glucopyranoside
Clc1c(c(cc(c1)Cl)C(=O)Nc2c(cc(cc2)OC)[N+](=O)[O-])O	Sigma-Aldrich	357.15	3,5-dichloro-2-hydroxy-N-(4-methoxy-2-nitrophenyl)benzamide
[N+](=O)([O-])c1cc(c(cc1)NC2CCC(CC2)O)C#N	Enamine	261.28	2-[(4-hydroxycyclohexyl)amino]-5-nitrobenzonitrile
[S](=O)(=O)(NC)c1cc(c(cc1)NC(C)c2cc3c(cc2)OCO3)[N+](=O)[O-]	Enamine	379.39	4-{[1-(1,3-benzodioxol-5-yl)ethyl]amino}-N-methyl-3-nitrobenzenesulfonamide
Fc1cc(c(cc1)NCc2nc([s]c2)NC)[N+](=O)[O-]	Enamine	282.3	N-{4-[(4-fluoro-2-nitroanilino)methyl]-1,3-thiazol-2-yl}-N-methylamine
Clc1c(cc(c(c1)[N+](=O)[O-])NCC(O)C(O)C(O)CO)CC	Sigma-Aldrich	334.76	1-(4-chloro-5-ethyl-2-nitroanilino)-1-deoxypentitol
[N+](=O)([O-])c1c(ccc(c1)C(=O)C)NCC2N(CCC2)C	Enamine	277.32	1-(4-{[(1-methyl-2-pyrrolidinyl)methyl]amino}-3-nitrophenyl)ethanone
[s]1c(nc(c1)C)Sc2c(cc(cc2)[N+](=O)[O-])C(=O)O	Enamine	296.33	2-[(4-methyl-1,3-thiazol-2-yl)sulfanyl]-5-nitrobenzoic acid
[N+](=O)([O-])c1c(ccc(c1)C(=O)C)NCCc2ccc(cc2)OC	Enamine	314.34	1-(4-{[2-(4-methoxyphenyl)ethyl]amino}-3-nitrophenyl)ethanone
FC(F)(F)c1cc(c(cc1)NCC(CO)Cc2[o]ccc2)[N+](=O)[O-]	Enamine	344.29	3-(2-furyl)-2-{[2-nitro-4-(trifluoromethyl)anilino]methyl}-1-propanol
S(c2nc(cc(n2)O)O)c1c(cc(cc1)C(=O)N)[N+](=O)[O-]	Labotest	308.27	4-[(4,6-dihydroxy-2-pyrimidinyl)sulfanyl]-3-nitrobenzamide
FC(F)(F)c1cc(c(cc1)NCc2cc(c(cc2)OC)Cl)[N+](=O)[O-]	Key Organics / BIONET	360.72	N-(3-chloro-4-methoxybenzyl)-2-nitro-4-(trifluoromethyl)aniline
[N+](=O)([O-])c1c(ccc(c1)c3nc(n[o]3)C)NCC2OCCC2	ChemDiv	304.31	4-(3-methyl-1,2,4-oxadiazol-5-yl)-2-nitro-N-(tetrahydro-2-furanylmethyl)aniline
[S](=O)(=O)(C)c1cc(c(cc1)NCCc2ccccc2)[N+](=O)[O-]	Enamine	320.37	4-(methylsulfonyl)-2-nitro-N-(2-phenylethyl)aniline
S(c2nc(cc(n2)O)N)c1c(cc(cc1)C(=O)N)[N+](=O)[O-]	Labotest	307.29	4-[(4-amino-6-hydroxy-2-pyrimidinyl)sulfanyl]-3-nitrobenzamide
N(C2OC(C(C(C2O)O)O)CO)c1ncccc1	Vitas-M	256.26	N-(2-pyridinyl)hexopyranosylamine

O1[C@H]([C@@H]([C@H]([C@@H]([C@H]1CO)O)O)O)Oc2ccccc2	Sigma-Aldrich	256.26	phenyl beta-D-glucopyranoside
[S](=O)(=O)(OC)O.N2(C=CC(=N)N(C2=O)C)[C@@H]1O[C@@H]([C@H]([C@H]1O)O)CO	Sigma-Aldrich	369.35	1-((2R,3R,4S,5R)-3,4-dihydroxy-5-(hydroxymethyl)tetrahydrofuran-2-yl)-4-imino-3-methyl-3,4-dihydropyrimidin-2(1H)-one methyl sulfate
Brc1ccc(cc1)N[C@@H]2OC[C@H](C(C2O)O)O	Vitas-M	304.14	N-(4-bromophenyl)-beta-D-glycero-pentopyranosylamine
[N+](=O)([O-])c1cc(ccc1)NC2OC(C(C2O)O)CO	Vitas-M	270.24	N-(3-nitrophenyl)pentofuranosylamine
S([C@@H]2O[C@@H]([C@H]([C@@H]([C@H]2O)O)O)CO)c1ccc(cc1)C	Sigma-Aldrich	286.35	4-methylphenyl 1-thio-beta-D-glucopyranoside
[N+](=O)([O-])c1ccc(cc1)O[C@@H]2O[C@H]([C@@H]([C@H]2O)O)CO	Sigma-Aldrich	271.23	4-nitrophenyl alpha-L-arabinofuranoside
